# Trends in hypertension prevalence, awareness, treatment, and control: an 8-year follow-up study from rural North India

**DOI:** 10.1038/s41598-023-37082-4

**Published:** 2023-06-19

**Authors:** Imnameren Longkumer, Suniti Yadav, Sunanda Rajkumari, Kallur Nava Saraswathy

**Affiliations:** Laboratory of Biochemical and Molecular Anthropology, Department of Anthropology, University of Delhi, Delhi, 110007 India

**Keywords:** Public health, Population screening

## Abstract

Hypertension is a major contributor to global CVD burden. LMICs including India is challenged with rising hypertension prevalence, yet limited studies are available on temporal change and incidence among community-cohorts. This study aimed to describe trends in hypertension prevalence, awareness, treatment, and control over 8 years among a rural community-cohort from Haryana, India. The study also lends towards an analysis of incidence. Adults ≥ 30 years (N = 1542) recruited during baseline cross-sectional study between 2011 and 2014 were followed up after a median 8.1 years. At endline, demographic/lifestyle characteristics and blood pressure were re-examined. Overall median SBP significantly increased from 120 mmHg at baseline to 125.5 mmHg at endline (*p* < 0.001), while hypertension prevalence increased from 34.4% (95% CI 32.0–36.9) to 40.4% (95% CI 37.5–43.4) (*p* = 0.002). Age-standardized hypertension incidence was 30.2% (95% CI 26.7–35.2) over 8 years. Among hypertensive group, awareness, treatment, and control increased from 9.6, 8.8 and 5.0% to 31.8, 27.3 and 9.6% (*p* < 0.05), respectively. Increasing trend in SBP and hypertension prevalence was observed as the cohort ages. This increase is supported by the high incidence of hypertension. Nevertheless, our study highlights positive trends in hypertension care cascade but poor control, suggesting that this trend may not be adequately impactful to reduce hypertension burden.

## Introduction

Hypertension has invariably emerged as the leading risk factor for stroke and cardiovascular diseases (CVDs)^1–3^. The latest NCD Risk Factor Collaboration study revealed that the global trend in absolute number of hypertensive individuals doubled between 1990 and 2019^4^. However, global hypertension prevalence remained stable—32% in 1990 and 33% in 2019^4^, mainly due to the declining trend of hypertension prevalence in high-income countries (HICs)^5,6^. In contrast, an increasing trend in hypertension prevalence is still observed in lower-middle-income countries (LMICs)^5–7^. By 2019, it has been estimated that more than 1 billion people were living with hypertension in LMICs, which accounts for 82% of the global population with hypertension^4^.

Like most LMICs, India has been experiencing a drastic epidemiological transition for the past two decades, with a major shift of disease burden from communicable to non-communicable^8^. Today, more than 60% of the nation’s total deaths are due to NCDs, of which stroke and CVDs constitute almost 30%^8,9^. Considering this, hypertension has emerged as a major disease burden, contributing to increased mortality^10^. Compared to earlier report^11^, hypertension prevalence in India has shown an increasing trend during the past two decades^12,13^, with most recent national estimates of up to 30%^14,15^. However, these studies estimated hypertension prevalence using cross-sectional study design thus, limiting the establishment of its temporal change within each cohort. Moreover, there are limited studies from India investigating the incidence of hypertension among community cohorts^16–20^. Thus, follow-up study designs will be more precise and reliable to estimate changing trend within the same cohort over time.

Further, contemporary to the rising hypertension prevalence, its care cascade including awareness, treatment, and control rates are rather poor in India, showing urban–rural disparity^15,21^. Similar results were also reported from the South Asian cohort of the Prospective Urban Rural Epidemiology (PURE) study^22^ with low awareness, treatment, and control rates, respectively among hypertensive group in both urban (45.9%, 37.6% and 15.4%) and rural (32.5%, 23.6% and 9.3%) areas. This trend improved only in individuals above 45 years and older, showing 55.7% awareness, 38.9% on treatment and 31.7% controlled blood pressure (BP) in India^23^. Even so, very few studies in India have reported temporal change in hypertension awareness, treatment and control using repeat cross-sectional studies^24,25^.

The Indian state of Haryana belongs to higher-middle epidemiological transition level (ETL)^8^. Under this ETL, 34% of total deaths were attributed to CVDs, while Haryana alone constituted a higher than national average rate of ischemic heart disease^8,10^. Moreover, high BP was one of the major risk factors for ischemic heart disease and stroke^10^. A recent study by Thakur and Nangia^26^ in Haryana reported prevalence of hypertension (26.2%), awareness (33.4%), treatment (26.3%) and control (12%) using a cross-sectional study design. However, till date, there is no prospective study investigating the changing trends in hypertension prevalence, its treatment cascade, and estimating incidence of hypertension in Haryana. Thus, to fill this knowledge gap, the present study assessed the temporal change in hypertension prevalence, awareness, treatment, and control over eight years among a community cohort residing in a rural area of Haryana, North India. The study also lends towards an analysis of incidence of hypertension among the same cohort.

## Materials and methods

### Study participants and formation of the cohort

**Figure 1:**
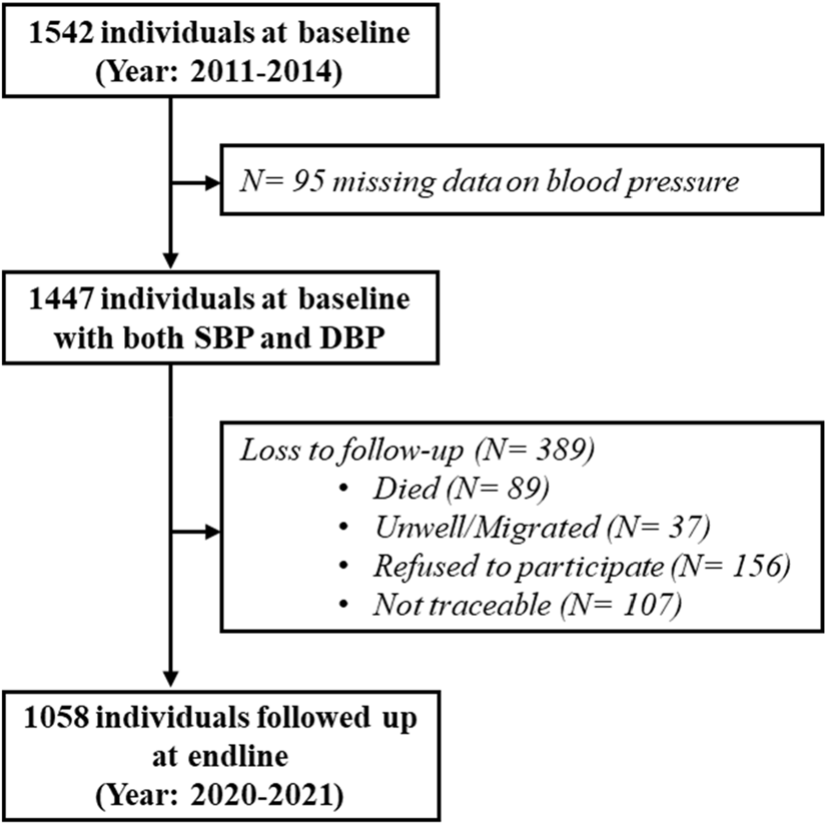
Flowchart illustrating the formation of the study cohort.

The present study was an extension of a previous cross-sectional study. This baseline study was conducted between October 2011 and April 2014 to assess cardiovascular risk factors among a rural community from Haryana, North India. The selected community belong to a Mendelian population and follow caste endogamy, village, surname and *gotra* exogamy and share a common gene pool with similar lifestyle and socio-cultural practices. They are predominantly agriculturists and follow lacto-vegetarian diet. Initially a pilot survey was conducted to identify district, blocks, and villages with highest density of the selected community. Subsequently, fieldwork was conducted in Palwal district across two blocks i.e., Hathin and Hodal and included fifteen villages. A total of 1542 individuals aged ≥ 30 years were recruited at baseline through door-to-door household survey. As part of the present endline study, participants from the baseline study were followed up after a median of 8.1 years (range: 6.8 to 9.6 years) between October 2020 and March 2021. The formation of study cohort from baseline to endline is described in Fig. 1. Of the total 1542 individuals at baseline, 95 individuals had missing data on BP. Thus, 1447 individuals were targeted to be followed up at endline. Of these included individuals, 389 (26.9%) individuals were loss to follow-up. The reasons were: death (N = 89), unwell or migration (N = 37), refusal to participate at endline examination (N = 156), and not traceable (N = 107). Finally, one thousand fifty-eight (73.1%) individuals were successfully followed up and included in the present endline analysis.

### Ethics approval

Both baseline and endline investigations were conducted following the principles of the Declaration of Helsinki and Indian Council of Medical Research guidelines for biomedical studies involving human subjects. Moreover, both investigations were approved by the Institutional Ethics Committee, Department of Anthropology, University of Delhi. Prior to recruitment and data collection, all participants provided written and informed consent (transcribed in local language).

### Data collection

Demographic and lifestyle data were collected using a pre-tested and modified interview schedule, administered separately in both baseline and endline. The information included age, sex, marital status, education level, occupation, smoking (*beedi* i.e., hand-rolled cigarettes and/or *hookah* i.e., water pipe), alcohol consumption, and dietary habit. In both the studies, all investigators were trained by the same instructor for administration of schedule and selection of correct cuff size for blood pressure measurement. A mercury sphygmomanometer was used at baseline to measure BP, while an automated BP measuring device (model HEM-7124, Omron Corporation, Tokyo, Japan) was used at endline. In both the studies, investigators recorded three consecutive BP readings measured from the right arm of each participant, seated upright after having rested for at least 10 min. Moreover, participants were informed not to smoke *beedi* and/or *hookah*, drink tea/coffee or perform any physical activity 30 min prior to BP measurement. Accordingly, the average of the three BP readings were considered as the final.

### Definitions

In both baseline and endline, hypertension was defined based on the standard recommendations of the World Hypertension League Expert Committee^27^ as systolic blood pressure (SBP) ≥ 140 mmHg or diastolic blood pressure (DBP) ≥ 90 mmHg or the use of antihypertensive medications. Awareness was defined as participants who were previously been diagnosed with hypertension by a health professional, or were taking antihypertensive medication. Treatment was defined as the proportion of individuals currently on antihypertensive medication or use of it during the last 3 months. Hypertension control was defined as the proportion of individuals with current SBP < 140 mmHg and DBP < 90 mmHg^15^.

### Statistical analysis

Change in socio-demographic and lifestyle characteristics were summarized using frequency and percentage. Normality test for continuous variables was performed and median values were considered when the distribution was not normal. Continuous variables were expressed by median (interquartile range) and categorical variables were expressed by number (percentage). Comparisons between groups used the Mann–Whitney *U* test for continuous variables and chi-square test for categorical variables. Age-standardization for incidence of hypertension was calculated according to the direct method and using 2011 Indian census data as standard population. The Mantel–Haenszel test was used for analysing trends in hypertension prevalence from baseline to endline. Change in BP from baseline to endline was represented using Kernel-Density Plot (KDP) and median difference was calculated between baseline and endline. Proportion of awareness, treatment and control were estimated from the hypertensive group in both baseline and endline, and the inter-study differences were calculated using Pearson chi-square test. Statistical significance at *p* < 0.05 (two-sided) was considered for all tests. Data analysis was performed using SPSS version 22.0 (IBM SPSS Statistics for Windows, Version 22.0. Armonk, NY: IBM Corp).

## Results

### Socio-demographic and lifestyle characteristics at baseline and endline (Table 1)

**Table 1:** Change in socio-demographic and lifestyle characteristics among the studied cohort from baseline to endline.

Variables	Baseline	Endline	*p* value
Age			
Median (IQR)	46.0 (40.0–55.0)	54.3 (48.0–62.9)	** < 0.001**
Sex, N (%)			
Females	1063 (68.9)	788 (71.1)	0.22
Males	479 (31.1)	320 (28.9)	
Marital status, N (%)			
Married	1315 (91.7)	967 (87.9)	** < 0.001**
Widow/widower	119 (8.3)	133 (12.1)	
Occupation, N (%)			
Primarily farming	1381 (90.6)	938 (86.0)	** < 0.001**
Private/government job	114 (7.5)	33 (3.0)	
Retired/dependant	30 (1.9)	120 (11.0)	
Educational level, N (%)			
Non-literate	827 (55.2)	687 (62.9)	** < 0.001**
Literate	670 (44.8)	405 (37.1)	
Dietary habit, N (%)			
Vegetarian	1381 (93.2)	1092 (99.5)	** < 0.001**
Non-vegetarian	101 (6.8)	5 (0.5)	
Alcohol consumption, N (%)			
No	1421(92.3)	889 (89.1)	**0.005**
Yes	118 (7.7)	109 (10.9)	
Smoking status, N (%)			
No	714 (48.8)	502 (47.4)	0.47
Yes	749 (51.2)	558 (52.6)	

Significant values are in bold.

Median age of the population increased significantly from baseline [46.0 (IQR: 40.0–55.0)] to endline [54.3 (IQR: 48.0–62.9)]. Moreover, the demographic characteristics that increased significantly from baseline to endline were widow or widower (8.3% at baseline vs. 12.1% at endline; *p* < 0.001) and retired or dependant individuals (1.9% at baseline vs. 11.0% at endline; *p* < 0.001). On the other hand, there was a significant difference in the distribution of educational status between the two studies. With respect to lifestyle variables, the present endline study observed a significant decrease in individuals consuming non-vegetarian food (6.8% at baseline vs. 0.5% at endline; *p* < 0.001). Conversely, individuals consuming alcohol were significantly higher in the present endline study (10.9%) as compared to baseline study (7.7%) (*p* = 0.005).

### Hypertension prevalence and incidence over eight years (Table 2)

**Table 2:** Prevalence and incidence of hypertension in the study cohort.

	Baseline		Endline		% difference	*p* value^a^	Endline	
	N	Prevalence, % (95% CI)	N	Prevalence, % (95% CI)			N	Incidence,^b^ % (95% CI)
Total	1447	34.4 (31.5–37.6)	1058	40.4 (37.7–44.5)	6	**0.002**	684	30.2 (26.7–35.2)
Sex								
Females	1012	31.0 (27.7–34.7)	755	37.0 (32.7–41.6)	6	**0.01**	514	28.1 (23.6–33.1)
Males	435	42.3 (36.4–48.9)	303	49.2 (41.6–57.7)	6.9	0.07	170	39.5 (30.0–39.4)

^a^Statistical comparisons for the trend of prevalence from baseline to endline are from Mantel–Haenszel test.

^b^Standardized incidence at endline was age-adjusted for overall and sex-specific.

Significant values are in bold.

The overall prevalence of hypertension significantly increased by 6% over 8 years from baseline [34.4% (95% CI 32.0–36.9)] to endline [40.4% (95% CI 37.5–43.4)] (*p* = 0.002). Similar significant increase was observed among females showing 6% increase over 8 years from baseline [31.0% (95% CI 27.7–34.7)] to endline [37.0% (95% CI 32.7–41.6)] (*p* = 0.01). The prevalence of hypertension among males increased by 6.9% from baseline [42.3% (95% CI 36.4–48.9)] to endline [49.2% (95% CI 41.6–57.7)], albeit with no statistical significance (*p* = 0.07). The overall incidence of hypertension at endline was estimated from baseline non-hypertensive individuals (SBP < 140 mmHg and DBP < 90 mmHg). From a total of 949 non-hypertensive individuals at baseline, 684 individuals were successfully followed up and their BP was measured. The age-standardized incidence of hypertension over 8 years was 30.2% (95% CI 26.7–35.2), yielding an annual hypertension incidence of 3.7% in the study cohort. Moreover, hypertension incidence was significantly higher among males [39.5% (95% CI 30.0–39.4)] as compared to females [28.1% (95% CI 23.6–33.1)] (*p* = 0.008).

### Change in blood pressure over eight years

**Figure 2:**
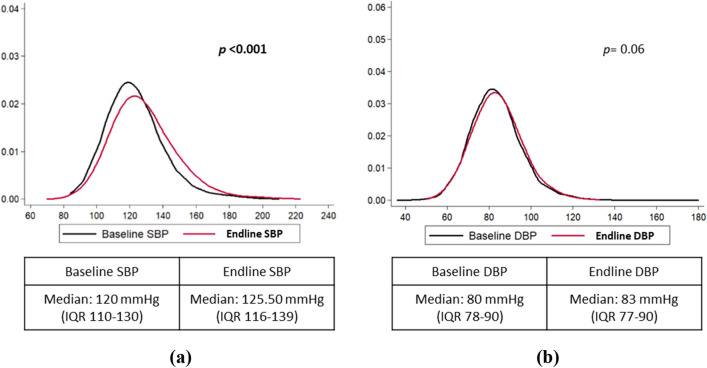
(**a**) Panel 1—KDP showing the change in SBP from baseline to endline; Panel 2—Median change in SBP from baseline to endline. (**b**) Panel 1—KDP showing the change in DBP from baseline to endline; Panel 2—Median change in DBP from baseline to endline. *KDP* kernel density plot; *SBP* systolic blood pressure, *DBP* diastolic blood pressure; *IQR* interquartile range.

There was an overall significant increase in SBP in the study cohort showing median change from 120 mmHg (IQR 110–130) at baseline to 125.50 mmHg (IQR 116–139) at endline (*p* < 0.001) [Fig. 2 (a)]. Alternately, median DBP increased from 80 mmHg (IQR 78–90) at baseline to 83 mmHg (IQR 77–90) at endline, albeit with no statistical significance (borderline significance at *p* = 0.06) in the study cohort [Fig. 2 (b)]. With respect to change in BP among hypertensive group (Table 3), median SBP significantly increased from baseline (137 mmHg) to endline (142 mmHg) (*p* < 0.001) and among both males [(baseline: 138 mmHg vs. endline: 145 mmHg), *p* < 0.001)] and females [(baseline: 135 mmHg *vs.* endline: 141.5 mmHg), *p* < 0.001)]. Nonetheless,  there was no significant change in median DBP from baseline to endline among hypertensive group.

**Table 3:** Change in median systolic and diastolic blood pressure from baseline to endline among hypertensive individuals.

	Baseline, median (IQR)	Endline, median (IQR)	*p* value
Total			
SBP	137.00 (128.00–146.00)	142.00 (131.16–152.00)	** < 0.001**
DBP	92.00 (90.00–100.00)	91.50 (87.50–98.38)	0.10
Females			
SBP	135.00 (128.00–144.00)	141.50 (130.00–151.00)	** < 0.001**
DBP	90.00 (90.00–98.00)	91.00 (86.00–98.00)	0.08
Males			
SBP	138.00 (130.00–148.00)	145.00 (135.25–154.00)	** < 0.001**
DBP	92.00 (90.00–100.00)	91.50 (88.25–99.75)	0.32

*SBP* systolic blood pressure, *DBP* diastolic blood pressure, *IQR* interquartile range. Statistical comparisons for the change in blood pressure from baseline to endline are from Mann–Whitney *U* test.

Significant values are in bold.

### Change in hypertension awareness, treatment, and control over eight years (Table 4)

**Table 4:** Change in hypertension awareness, treatment, and control among hypertensive group from baseline (N = 498) to endline (N = 428).

	Awareness, N (%)		*p* value	Treatment, N (%)		*p* value	Control, N (%)		*p* value
	Baseline	Endline		Baseline	Endline		Baseline	Endline	
Total	48 (9.6)	136 (31.8)	** < 0.001**	44 (8.8)	117 (27.3)	** < 0.001**	25 (5.0)	41 (9.6)	**0.006**
Females	33 (10.5)	106 (36.2)	** < 0.001**	32 (10.2)	96 (32.8)	** < 0.001**	20 (6.4)	35 (11.9)	**0.016**
Males	15 (8.2)	36 (23.4)	** < 0.001**	12 (6.5)	25 (16.2)	**0.004**	5 (2.7)	8 (5.2)	0.238
*p* value	0.39	**0.006**		0.16	** < 0001**		0.07	**0.02**	

Significant values are in bold.

Awareness among hypertensive group increased from 9.6% at baseline to 31.8% at endline (*p* < 0.001). Moreover, this increase was significant among both males (8.2% at baseline vs. 23.4% at endline; *p* < 0.001) and females (10.5% at baseline vs. 36.2% at endline; *p* < 0.001). Although there was no sex-wise difference in hypertension awareness at baseline (females: 10.5% vs. males: 8.2%; *p* = 0.39), females were more aware of their hypertensive status as compared to males at endline i.e., 36.2% and 23.4%, respectively (*p* < 0.001).

Treatment among hypertensive group increased significantly from 8.8% at baseline to 27.3% at endline (*p* < 0.001). This was true among both males (6.5% at baseline vs. 16.2% at endline; *p* = 0.004) and females (10.2% at baseline vs. 32.8% at endline; *p* < 0.001). There was no sex-wise difference in treatment at baseline (females: 10.2% vs. males: 6.5%; *p* = 0.16). However, this trend changed significantly at endline with higher prevalence of hypertension treatment among females (32.8%) as compared to males (16.2%) (*p* < 0.001).

The control of hypertension (current SBP < 140 mmHg and DBP < 90 mmHg) among hypertensive group significantly increased from 5.0% at baseline to 9.6% at endline (*p* = 0.006). Similar significant trend was observed among females showing almost 50% increase in hypertension control from 6.4% at baseline to 11.9% at endline (*p* = 0.016). However, no such significant trend was observed among males (*p* = 0.238). Further, no sex-wise difference in hypertension control was observed at baseline (females: 6.4% vs. males: 2.7%; *p* = 0.07), although this trend changed significantly at endline with hypertension control higher among females (11.9%) as compared to males (5.2%) (*p* = 0.02).

**Figure 3:**
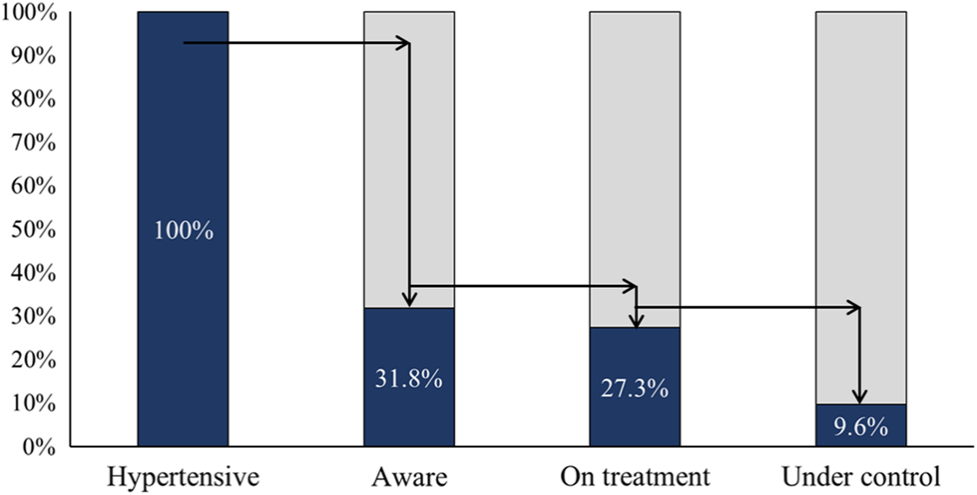
Endline awareness, treatment, and control among hypertensive group.

Nonetheless, endline awareness, treatment and control among hypertensive group is also depicted in Fig. 3. Of the total 428 (40.4%) hypertensives at endline, 136 (31.8%) were aware of their hypertension status, 117 (27.3%) were under treatment for hypertension, and only 41 (9.6%) had their BP under control.

## Discussion

Our analysis on temporal change in hypertension prevalence among a rural community from North India revealed significant increase from 34.4% at baseline to 40.4% at endline over 8 years. This increase is corroborated by the high age-standardized incidence of hypertension (30.2%) in the same cohort. Our study is consistent with population-based longitudinal studies showing rising trend of hypertension prevalence with increasing age^28^. Nonetheless, this trend is accompanied by a poor hypertension control suggesting mounting hypertension burden in this population.

In India, several studies have reported rising trend in hypertension prevalence over the past decade^12,13,29,30^. Moreover, hypertension prevalence observed at endline is comparable to most urban populations^22,23,30,31^. Previous reports have indicated that the rate at which hypertension prevalence increases was higher in rural population as compared to urban population^24,32^. This trend is also supported by a recent meta-analysis of studies from 66 LMICs^33^. Currently, India is experiencing an urban–rural convergence in hypertension prevalence^32^. The rising hypertension prevalence in our study cohort over eight years hint towards this urban–rural convergence. Further, there have been only few community-based studies from India investigating the incidence of hypertension. A previous study from rural Kerala reported 23.6% incidence of hypertension over 7 years^16^. Recently, the Chennai Urban Rural Epidemiology Study reported an overall hypertension incidence of 25.8% over 9 years^19^. Our study, with similar follow-up duration, observed higher incidence of hypertension. This may have driven the increased prevalence of hypertension from baseline to endline in our study cohort.

Studies have indicated that urbanization of rural populations with consequent modification in lifestyle and dietary behaviours have brought about epidemiological transition^33–35^. The present study cohort resides in villages under Palwal district of Haryana, which is near Delhi-NCR (urban cities). Hence, the community is exposed to most semi-urban or urban lifestyles, particularly high intake of calorie-dense foods, fats, and salt^36,37^ which might have influenced the increase in BP^32,38^. Further, as the cohort ages, we found a significant increase in individuals retiring from their agricultural work. This shift from an active physical occupation to almost sedentary lifestyle may have attributed to the rising BP^39^. Nonetheless, since the participants from our cohort comprised of adults > 30 years at baseline, the significant increase in BP, particularly SBP (both overall and in hypertensive group), may have been due to age-related arterial vascular thickening/stiffening resulting in higher SBP output, while the same process is associated with decreased DBP^40^.

Besides, contemporary risk factors like air pollution may be playing a significant role in increasing BP and hypertension prevalence/incidence among this community. A seminal study by Prabhakaran et al.^41^ from urban Delhi demonstrated a significant temporal association between long-term exposure to ambient air pollution (PM_2.5_) and increased SBP, and incident hypertension. Considering that our study area and Delhi are adjacent, it is plausible that exposure to ambient air pollution might trigger the rise in BP. Additionally, several studies reported significant association of both ambient and household air pollution (use of kerosene or solid fuels for cooking) with hypertension, CVDs, and mortality, particularly in LMICs^42,43^. At baseline, more than 98% of individuals in our cohort were exposed to household/indoor air pollution due to the use of biomass (firewood, dried cow-dung cake, agricultural residue, etc.) as cooking fuel (data not shown in table). Since mitigating the effects of air pollution are less subjected to individual-level control, reducing both ambient and household air pollution requires the involvement of government policies and activation of public health systems to provide access to clean air and clean fuel.

Further, our analysis on awareness, treatment and control among hypertensive group showed significant increase from baseline to endline. Similar results were reported by Roy et al.^24^ and Gupta et al.^25^ using repeat cross-sectional study design in rural and urban populations, respectively. Although not as robust as in HICs, hypertension treatment and control rates have been improving steadily in LMICs since 1990^4^. Our study also supports the improving trend in awareness, treatment, and control rates among rural populations. However, the magnitude at which these rates improve is inadequate to have an impact in decreasing hypertension prevalence. Studies have estimated these rates in rural India to be between 25–27%, 13–25%, and 9–13%, respectively^12,15,21,22,44^. In comparison to these studies, our study showed slightly better awareness and treatment rates, however, the control rate was worse. Thus, the highest loss in hypertension care cascade has been in the control rate, and our study participants performed the poorest as compared to reports from rural India^12,15,21,22^.

Moreover, we found significant sex-wise difference in awareness, treatment, and control among hypertensive group at endline, whereby women were outperforming men in each component. However, this observation is not unique and has been reported in other studies from LMICs and India as well^45–48^. Possible reasons may include women attending antenatal care service wherein BP measurement is a core component, and fewer opportunistic screening of hypertension for men due to their general reluctancy in accessing health-care services.

Considering our study, it is imperative that hypertension management should be paramount. The cascade of hypertension care and control is influenced by several factors including policy-level, health system-level, community-level, and patient-level^12,49^. Results from the first phase of the India Hypertension Control Initiative (IHCI) has demonstrated significant improvement of hypertension control, with primary health-care facilities performing two times greater than secondary health-care facilities^50^. However, this initiative currently does not involve the private sector. Yet, in India, significant gaps in private–public primary and secondary care facilities have been documented^15^. Therefore, efforts should focus on strengthening public health-care facilities, especially improvement in the availability of essential medicines, technology, and medical personnel^51^. Further, studies have demonstrated effective hypertension control that involved the participation of community volunteers and health workers^52,53^. This highlights the strength on task-shifting of intervention for hypertension control from medical personnel to community members, thus opening a potential large-scale community-based intervention for treating hypertension.

Additionally, targeted population-based interventions are necessary for prevention and control of hypertension. Population intervention may include regulation of sodium directly through dietary practices as well as in food-products, improved nutrition strategies, and more stringent regulation on tobacco products. Also, the use of Mobile Medical Units in remote areas under the National Health Policy 2017 is a welcoming step^54^. However, the barriers for poor control rates and low awareness/treatment may be addressed through the local champions who can be trained to assist in hypertension awareness and link community to health systems^55^. National-level programmes addressing population-based interventions may thus be pivotal in improving the hypertension care cascade in controlling the burden of hypertension, especially in rural populations.

The strength of our study is the follow-up of the same community cohort over 8 years. Nonetheless, our study also presents some limitations: high attrition rate which has largely been influenced by the COVID-19 pandemic. Since our study is restricted to a single Mendelian community, it may not be generalizable up to the national level. Another limitation was the use of different instruments for measurement of BP at baseline and endline. This was unavoidable as the instruments used during baseline study was not available at the time of endline study. However, it has been demonstrated that compared to mercury sphygmomanometer, the currently used automated monitor underestimate BP^56^ and hence, the temporal change in BP would be higher among the study community.

## Conclusion

Our study observed significant increase in the prevalence of hypertension from baseline to endline over 8 years as the cohort ages. During the same period, the age-standardized incidence of hypertension was alarmingly high. Simultaneously, there was positive change in awareness, treatment, and control among hypertensive group. This favourable trend may be the outcome of our baseline study whereby hypertensive individuals were informed of their status and were encouraged to follow anti-hypertensive treatment regime. As such, focusing on population-based studies may provide opportunistic screening and early diagnosis of hypertension among community members that may necessitate them to adhere to hypertension care cascade. Nonetheless, this favourable trend is not adequate to reduce hypertension burden as the control rate is very poor. Hence, there is an urgent need to accelerate concerted efforts involving all stakeholders to (1) adopt preventive measures by promoting healthy lifestyle and (2) improve hypertension management, particularly focussing on interventions that will enhance the awareness, treatment, and control steps of hypertension care cascade.

## Data Availability

All data are presented in the manuscript. The datasets used to generate the results in the present study will be available from the corresponding author on a reasonable request.
